# miR-29 regulates metabolism by inhibiting JNK-1 expression in non-obese patients with type 2 diabetes mellitus and NAFLD

**DOI:** 10.1515/med-2023-0873

**Published:** 2023-12-06

**Authors:** Fangdong Lu, Huaping Gong, Linhai Duan, Yuan Yan, Long Chen, Pei Peng, Qiaoan Zhang, Wenrong Song, Jia Li

**Affiliations:** Endocrinology Department, Hanchuan People’s Hospital, Hanchuan 431600, China; Clinical Laboratory Department, Hanchuan People’s Hospital, Hanchuan 431600, China; Wuhan University of Science and Technology, Wuhan 430065, China; Endocrinology Department, Hanchuan People’s Hospital, No. 1 People’s Avenue, Hanchuan 431600, China

**Keywords:** miR-29, diabetes mellitus, NAFLD, JNK-1, AMPK signaling

## Abstract

Previous studies have shown that microRNAs (miRNAs) are involved in the regulation of a variety of metabolic diseases, which related to some important signal pathways. Our aim was to explore the possible mechanism of miRNAs by revealing the differential expression of serum miRNAs in different BMI of type 2 diabetes mellitus (T2DM) patients with non-alcoholic fatty liver disease (NAFLD). We found that miR-29 decreased liver aminotransferase gamma-GGT and uric acid levels by inhibiting the expression of JNK-1 in non-obese T2DM patients with NAFLD, and down-regulated the expression of atherosclerosis-related factor lipoprotein phospholipase A2 (Lp-PLA2). Combined with bioinformatics analysis, we speculate that this may be mediated by the AMPK signaling. These findings suggest that miR-29 may be a potential targeted therapeutic strategy in T2DM patients with NAFLD.

## 1 Introduction

Type 2 diabetes mellitus (T2DM) is a chronic, low-grade inflammatory disease characterized by insulin resistance and pancreatic beta cell dysfunction, the underlying molecular mechanism of which is still unclear. According to statistics, the number of diabetic patients worldwide will reach 783.2 million in 2045 [1]. Diabetes is often associated with obesity, heart disease, and non-alcoholic fatty liver disease (NAFLD). NAFLD is the most common liver disease in the world [2]. T2DM and NAFLD are closely related and promote each other, and the prevalence rate of the two is rising simultaneously. About 70–90% of patients with T2DM have NAFLD [3,4]. Diabetes is also one of the most decisive risk factors for accelerated progression of NAFLD to NASH, short for non-alcoholic steatohepatitis, is characterized by the accumulation of fat, inflammation, and liver cell damage, which can progress to liver fibrosis or cirrhosis [5,6]. They not only have common risk factors, but also aggravate target organ damage after co-morbidity, leading to insulin resistance, obesity, abnormal liver function, lipid metabolism disorder, hyperuricemia, atherosclerosis, and increasing the risk of poor prognosis in patients.

The potential mechanism of diabetes promotes the transition from NAFLD to NASH and fibrosis is still unclear [7,8]. In addition to insulin resistance, elevated liver enzymes, and vascular endothelial inflammation, there may be other factors involved in its regulation, including microRNAs (miRNAs). miRNAs are a class of single-stranded non-coding small RNA molecules, composed of about 20–22 nucleotides [9], which are involved in the regulation of gene expression and protein translation. Pretranslational regulation of miRNAs plays a key role in cell metabolism, fine-tuning metabolic homeostasis by targeting key rate-limiting enzymes in related pathways, and they have been shown to be associated with multiple metabolic diseases, including obesity, diabetes, NAFLD, and others. miRNAs may participate in the occurrence of T2DM combined with NAFLD through various signaling pathways, and play an unique role in aggravating diabetes complications and liver fibrosis. Our study found that the role of miR-29 in non-obese T2DM patients with NAFLD may improve hepatocellular inflammation, uric acid metabolism, and delay the process of atherosclerosis by inhibiting JNK-1.

miR-29 is an important miRNA, and the miR-29 family includes miR-29a, miR-29b, and miR-29c [10], which is important in a variety of biological processes. Studies indicate that miR-29 derived from beta cell exosomes is one of the major contributors to inflammatory signals in diabetes [11]. Recent studies have exposed that there is a close relationship between miR-29 and AMPK signaling. AMPK is an important energy sensor that regulates metabolism by phosphorylating key metabolic proteins and transcription factors, promoting energy catabolism and inhibiting energy storage pathways [12]. Activation of AMPK signaling can promote glucose uptake and oxidation, fatty acid oxidation, and mitochondrial biosynthesis, which have a profound impact on obesity-related diseases. JNK-1 is a protein kinase that belongs to the JNK family, which plays a key role in cell response to stress, inflammation, and apoptosis. The relationship between the JNK-I and AMPK signaling pathways can be complex. AMPK often acting to modulate or suppress JNK signaling, particularly in response to metabolic and stress-related challenges.

As we know, obesity is closely related to diabetes and NAFLD. In addition, a considerable number of non-obese T2DM patients with NAFLD in clinic [13,14], and the patients with BMI <25 kg/m^2^, the relevant research on whether there are differences in metabolic parameters between non-obese and obese T2DM with NAFLD is continuing to deepen. Whether there are differences in circulating miRNAs levels in T2DM patients with NAFLD in different BMI? Our study will take this issue into consideration to reveal the differential expression of miRNAs, changes in metabolism-related indicators, and possible signal pathways in T2DM patients with NAFLD of different BMI.

## 2 Materials and methods

### 2.1 Subjects

We enrolled patients from the Department of Endocrinology of Hanchuan People’s Hospital during the period from January 1, 2021 to February 1, 2022. A total of 83 T2DM patients combined with NAFLD were selected after evaluation, including 44 patients in the obese group (BMI ≥ 25 kg/m^2^) and 39 patients in the non-obese group (BMI < 25 kg/m^2^). About 5 mL of venous blood was collected from all patients after fasting for 12 h, centrifuged (4,000 rev/10 min) and stored at −80°C in a refrigerator.

### 2.2 Collection of general and clinical data

The height (m), weight (kg), and blood pressure (mmHg) of all patients were measured in a unified standard. Hemoglobin (HbA1c; Bio-Rad, D-10, China), platelet (PLT; Mindray, BC-6900, China), liver function (AST, ALT, and gamma-GGT) and renal function (UA and CysC) (Beckman CM, AU5800, Japan), cardiac phospholipase A2 (Lp-PLA2; Niukang, NTD-VO2, China), and other indicators (Table 1) were measured in the two groups. All procedures were approved by patients and the Ethics Committee of Hanchuan People’s Hospital (HCRY2021～001).

**Table 1:** Basic characteristics of study population

Characteristic	Obese (*n* = 44)	Non-obese (*n* = 39)
Age (years)	55.16 ± 11.88	58.97 ± 11.29
Male	25 (44)	17 (39)
BMI (kg/m^2^)	28.98 ± 2.34	22.92 ± 1.72*
HbA1c (%)	9.955 ± 1.95	10.4 ± 2.76
SBP (mmHg)	136.09 ± 17.80	134.87 ± 18.08
DBP (mmHg)	81.25 ± 14.67	81.44 ± 8.20
PLT (10^9^)	222.4 ± 59.56	214.2 ± 56.32

Data are *n* (%) or mean ± SD.

Only the BMI was significantly different between the groups.

### 2.3 Bioinformatics analysis

Two gene expression datasets (GSE51674 & GSE202167) were downloaded from the Gene Expression Omnibus (GEO) database. The GSE51674 microarray contained miRNA expression data from 12 T2DM patients and 4 healthy controls. The GSE202167 microarray contained miRNA expression data from three NAFLD patients and three control individuals. The significant differentially expressed genes (DEGs) were selected by the criterion of log2 (fold change) >1 and *P-*value <0.05. The target gene prediction tool miRWalk was used to predict the target genes of DEmiRNAs, and the target genes shared by more than three databases (miRDB, Targetscan, Mirtarbase) were screened. GO and KEGG enrichment analysis of target genes was performed using the DAVID database, and *P* < 0.05 was considered statistically significant. The mapping between the target genes (FUS, MMP9, IRS2, ICAM1, PTEN, LEP, FGF21, ADPN, and JNK1) and DEmiRNAs was imported into Cytoscape, and the miRNAs-mRNA network diagram was constructed.

### 2.4 Metabolic parameters detection

After thawing and centrifugation, all plasma samples were uniformly tested by ELISA to detect metabolism-related parameters, contained LEP (Bioswamp, cat. HM10684), ADPN (Bioswamp, cat. HM10728), FUS (Bioswamp, cat. HM12279), JNK-1 (Bioswamp, cat. HM13297), ICAM-1 (Bioswamp, cat. HM10945), PTEN (Bioswamp, cat. HM11386), FGF21 (Bioswamp, cat. HM11132), IRS-2 (Bioswamp, cat. HM13319), MMP9 (Bioswamp, cat. HM10095), and the levels of metabolic parameters in the two groups were compared.

### 2.5 Statistical analysis

All data are presented as the mean ± SEM. Data analysis involved unpaired Student’s *t*-test for two groups, as appropriate, using GraphPad Prism version 9. *P*＜0.05 was considered statistically significant.

## 3 Results

### 3.1 Gene expression profile and related pathways in T2DM patients with NAFLD

**Figure 1:**
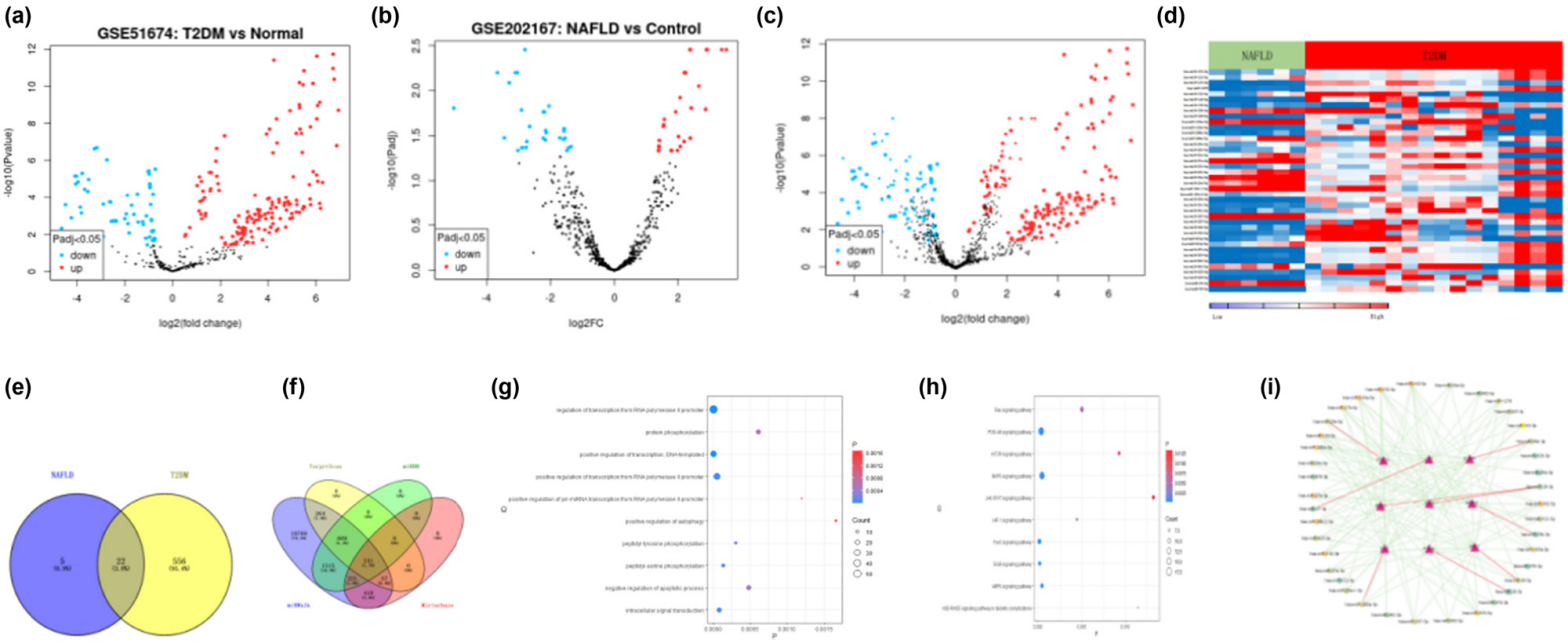
DEGs identification. (a–d) Volcano plots and heatmaps of the two GEO datasets (GSE51674 and GSE202167). The DEGs with |log FC| (fold change) >1 and *P* value <0.05 are shown. (e) Venn diagram of the two GEO datasets showed 22 shared genes (miR-122, miR-126, miR-1278, miR-142, miR-143, miR-150, miR-193a, miR-200a, miR-26b, miR-27a, miR-27b, miR-29a, miR-29b-1, miR-29b-2, miR-29c, miR-337, miR-342, miR-374a, miR-379, miR-582, miR-625, miR-98). (f) miRDB, Mirtarbase, miRwalk, and Targetscan databases were intermingled, and 1,128 target genes were obtained. (g and h) Top ten GO biological annotation processes and ten KEGG signaling pathways were screened with *P* < 0.05 as the criterion. (i) Cytoscape software exported the results of the miRNA–mRNA network of the miRNA-target gene.

A total of 578 DEmiRNAs were screened from GSE51674 microarray (Figure 1a) and 27 DEmiRNAs were screened from GSE202167 microarray (Figure 1b), and DEmiRNAs of the two microarrays were merged (Figure 1c); 22 miRNAs co-existing with NAFLD and T2DM were obtained, as shown in the heat map (Figure 1d). The intersection of DEmiRNAs of the two microarrays is shown in the Wynn diagram (Figure 1e). Multiple miRNA target gene prediction databases integrated with miRWalk were used to predict and collect target genes for DEmiRNAs, and the target genes in miRDB, Mirtarbase, and Targetscan databases were intermingled, and 1,128 target genes were obtained (Figure 1f). Gene oncology and KEGG pathway items were analyzed through selecting species of “human,” and the top ten GO biological annotation processes (Figure 1g) and ten KEGG signaling pathways (Figure 1h) were screened with *P* < 0.05 as the criterion. The obtained miRNAs co-associated combined T2DM with NAFLD, and nine target genes in this study were imported into Cytoscape software, and the miRNA-target gene regulatory network was constructed according to the targeting relationships, and the results of the miRNA–mRNA network were exported (Figure 1i).

### 3.2 Comparison of liver and kidney function, lipid metabolism, and cardiovascular risk between the two groups

**Figure 2:**
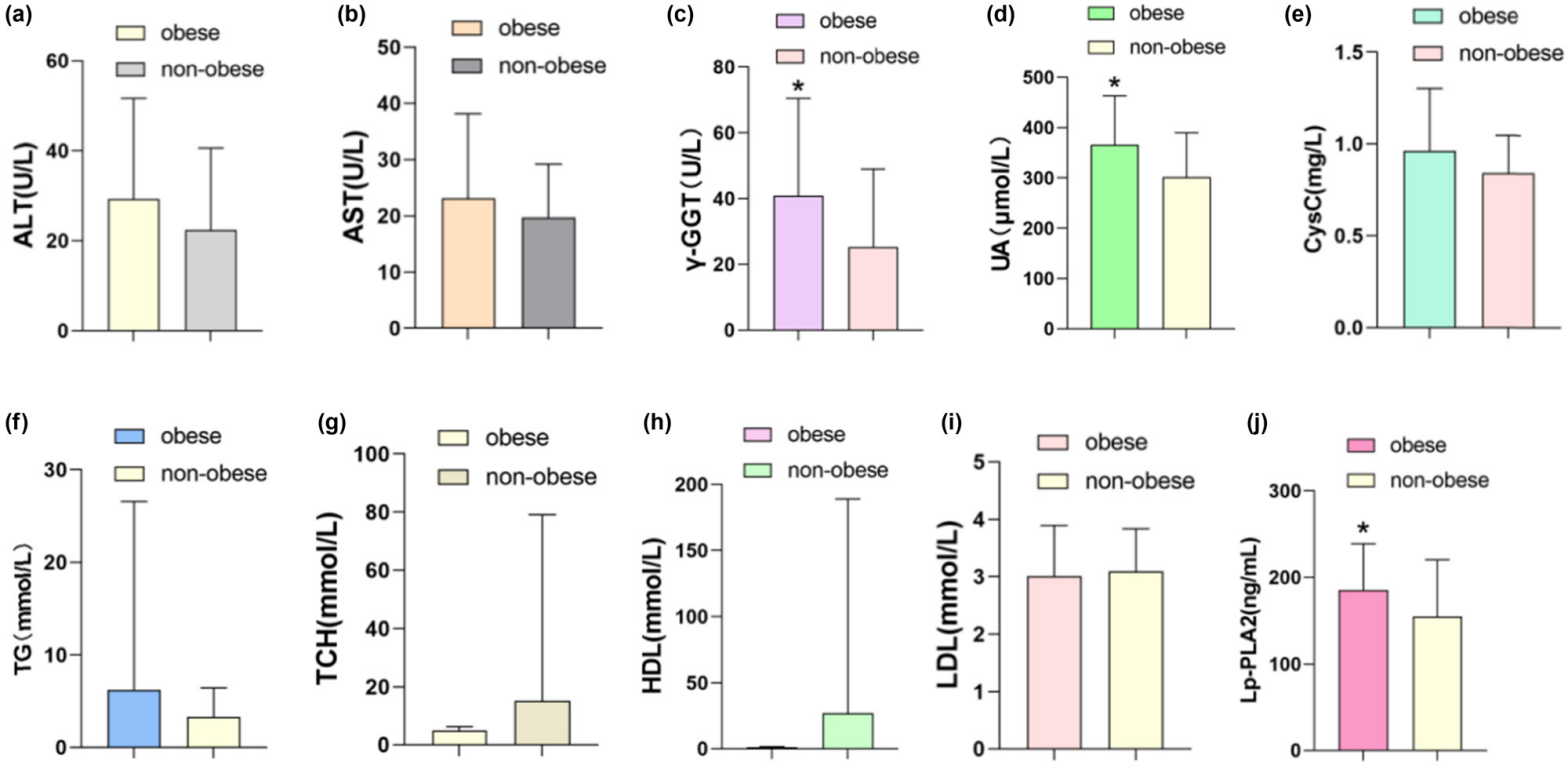
Liver function parameters ALT, AST, and γ-GGT in the two groups (a–c), γ-GGT in the non-obese group was significantly lower (*P* < 0.05). Renal function indicators of uric acid and cystatin C (d and e), uric acid in the non-obese group decreased significantly (*P* < 0.05). Lipid parameters of TG, TCH, HDL, and LDL (f–i), no significant difference between the two groups (*P* > 0.05). The cardiovascular risk factor Lp-PLA2 (j), was significantly decreased in the non-obese group (*P* < 0.05).

Liver function parameters contained ALT, AST, and γ-GGT. Our study found that γ-GGT in the non-obese group was significantly lower (Figure 2a–c), suggesting that hepatocyte injury and oxidative stress were more significant in the obese group. Renal function indicators contained uric acid and cystatin C (Figure 2d and e), this study confirmed that the level of uric acid in the non-obese group decreased significantly, indicating that the obese group had more serious disorders of uric acid metabolism. Lipid metabolism included TG, TCH, HDL, and LDL; to our surprise, there was no significant difference between the two groups (Figure 2f–i), implying that both groups had serious lipid metabolism disorders. The expression of Lp-PLA2, an indicator related to cardiovascular risk, was significantly decreased in the non-obese group (Figure 2j), referring to that weight loss can significantly improve the risk of cardiovascular atherosclerosis in T2DM patients with NAFLD.

### 3.3 Comparison of metabolic target genes expression between the two groups

**Figure 3:**
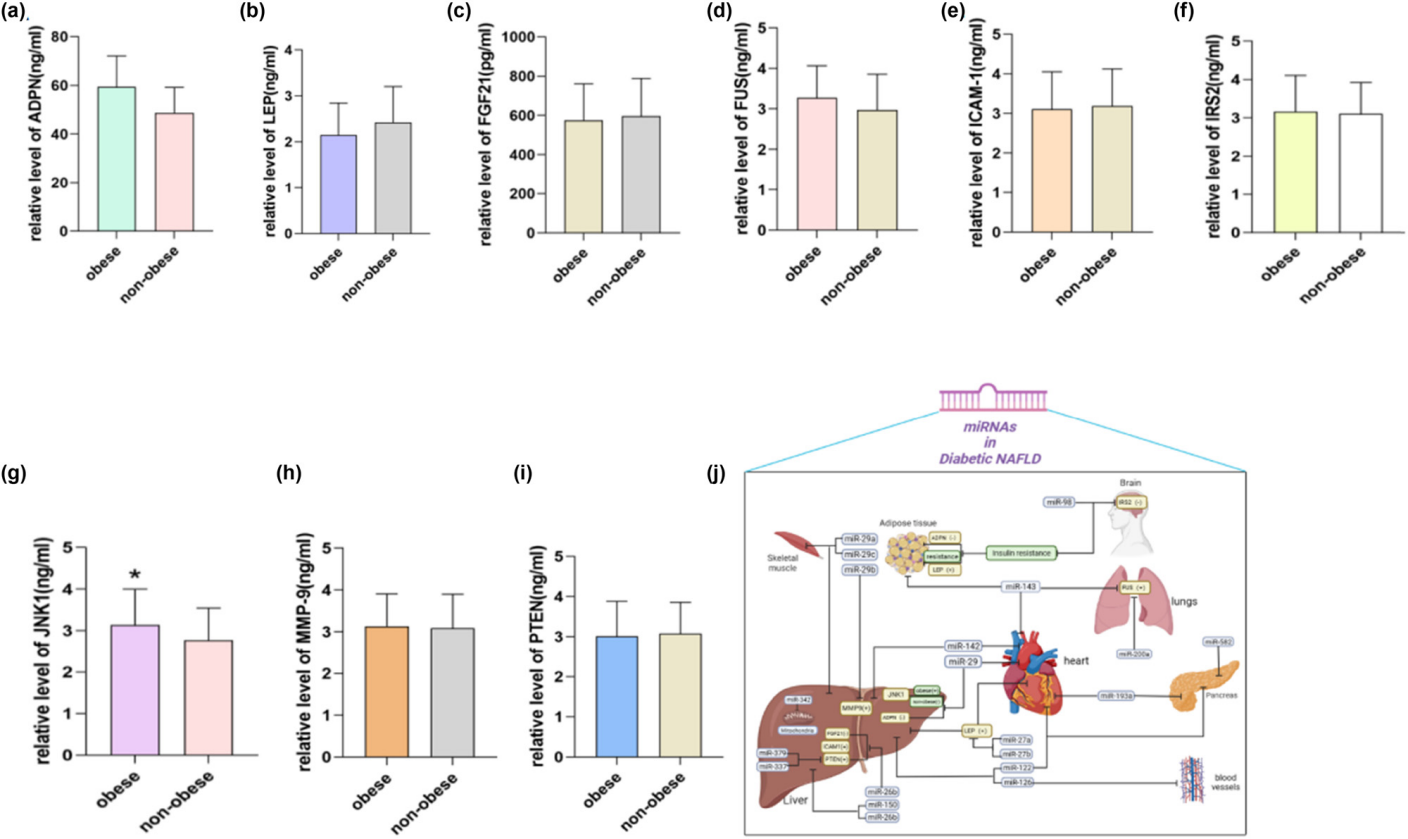
Metabolic factors FUS, MMP9, IRS2, ICAM1, PTEN, LEP, FGF21, ADPN, and JNK-1 were tested (a–i) (only JNK-1 was significantly different between the two groups (*P* < 0.05)). (j) miRNAs and the potential target genes in the regulation of T2DM patients with NAFLD.

We selected metabolic factors closely related to glycolipid metabolism, containing FUS, MMP9, IRS2, ICAM1, PTEN, LEP, FGF21, ADPN, and JNK-1 (Figure 3a–i). We found that only the expression of JNK-1 was significantly different between the two groups, and the expression of JNK-1 was decreased in the non-obese group. It hinted that the degree of inflammation and hepatocyte steatosis in the non-obese group was significantly improved compared to the obese group. Combining with bioinformatics analysis, we hypothesized that miR-29 was involved in regulating JNK-1 expression, which may be mediated by AMPK signaling, and it needs to be confirmed by further studies.

## 4 Discussion

Our work is the first to confirm that there are significant differences in metabolic markers in T2DM patients with NAFLD of different BMI, which have never been verified in previous work. Our study revealed that γ-GGT were significantly lower in non-obese T2DM patients with NAFLD than obese. γ-GGT is an important indicator of liver function, and its main role is to participate in amino acid metabolism. In NAFLD, the expression of γ-GGT is significantly increased due to the disorder of fat metabolism and hepatocyte injury [15]. Some studies have shown that γ-GGT levels were closely related to the severity and prognosis of NAFLD [16]. Therefore, we believe that obese T2DM patients with NAFLD have more serious liver fat accumulation and liver function impairment. In addition, some studies have also found that the level of γ-GGT in patients with NAFLD is highly correlated with metabolic syndrome, diabetes, and cardiovascular disease [17], indicating that γ-GGT may also be involved in the occurrence and development of these diseases. Therefore, it is necessary to closely monitor the level of γ-GGT in these patients, intervention and treatment in time, to delay the process of complications. At the same time, we found that patients in the non-obese group also had significantly lower uric acid levels than those in the obese group. We know that patients with NAFLD have impaired liver function and are unable to metabolize uric acid effectively, resulting in the accumulation of uric acid. At the same time, NAFLD patients are often accompanied by metabolic syndrome, T2DM, hypertension, and other metabolic diseases [18], all of them can lead to elevated uric acid levels. Our research shows that obese patients are more likely to be complicated with hyperuricemia. Uric acid is usually cleared by the liver and kidneys, and with the increase of BMI in patients with NAFLD, liver function may be abnormal, resulting in a decrease in the ability to clear uric acid, thus allowing uric acid to accumulate. In addition, patients with NAFLD may also affect the metabolism of uric acid due to metabolic disorders and inflammatory responses [19], increasing the risk of cardiovascular disease. Therefore, the management of uric acid level in patients with NAFLD is an important clinical issue. Particularly in NAFLD patients with higher BMI, uric acid should be strictly controlled and treated as soon as possible to delay the occurrence of cardiovascular and cerebrovascular diseases. Our study shows that the liver and kidney function impairment in non-obese patients is lower than that in the obese group. Therefore, weight loss is still an urgent need for T2DM patients with NAFLD.

Lp-PLA2 is an enzyme involved in cell membrane phospholipid metabolism, which has a decisive impact on metabolic tissues. It can catalyze the hydrolysis of phosphatidylcholine into phosphatidylic acid and free choline, and participate in various physiological and pathological processes [20]. We found that Lp-PLA2 was also significantly reduced in the non-obese group. Lp-PLA2 may be the crucial factor of NAFLD, several studies have shown that the expression of Lp-PLA2 in NAFLD is elevated, and its high expression may be related to the process of NAFLD. A mouse study showed that the expression of Lp-PLA2 was significantly increased in a high-fat diet-induced mouse model of NAFLD [21]. Another study found that Lp-PLA2 expression was also significantly increased in human NAFLD tissue [22]. In addition, Lp-PLA2 can also stimulate inflammatory response [23], which may aggravate hepatocyte inflammation in patients with NAFLD. The role of Lp-PLA2 in metabolic tissue mainly includes participating in inflammatory reaction by releasing bioactive substances such as free fatty acids and lysophosphatidylcholine [24], regulating apoptosis and inducing platelet aggregation, thus participating in the physiological and pathological processes of thrombosis [25], which is a key risk factor of atherosclerosis. Our study suggests that the risk of atherosclerosis is lower in the non-obese group than in the obese group. Lp-PLA2 may be a vital factor in the process of NAFLD, more studies could further explore the mechanism of Lp-PLA2 and ensure its potential as a therapeutic target in NAFLD.

By combining bioinformatics analysis with clinical data, we found that a variety of miRNAs may be involved in the regulation of T2DM with NAFLD, and we predicted some possible signaling and target genes (Figure 3j). We found that JNK-1 expression was significantly different between the two groups. JNK-1, a protein kinase, is expressed in many metabolic tissues, especially in liver, adipose tissue, and islet cells [26]. JNK-1 is an important signal transduction molecule that can mediate cell response to environmental stimuli and regulate a few downstream target genes through phosphorylation, thereby affecting the growth, differentiation, and death of cells and playing a unique role in many diseases, such as diabetes, cancer, and neurodegenerative diseases [27]. Studies showed that excessive activation of JNK-1 in the liver could lead to liver injury, liver fibrosis, liver cancer, and other diseases [28]. The activation of JNK-1 in adipose tissue can lead to the apoptosis of adipocytes and the disorder of fat metabolism, which leads to the occurrence of obesity and metabolic diseases. In addition, excessive activation of JNK-1 in islet cells can lead to insufficient insulin secretion and apoptosis of islet cells, thus leading to diabetes [29]. Our study indicated that the expression of JNK-I in non-obese patients was significantly lower, which may indirectly imply that non-obese patients with T2DM with NAFLD have improved lipid metabolism and insulin resistance related parameters compared to obese patients; therefore, weight loss is a key measure for obese T2DM patients with NAFLD. The signal pathway of glycolipid metabolism involved by JNK-1 is very complex, including cross-regulation, and feedback mechanisms of multiple signalings contain AMPK signaling, NF-kB signaling, ROS signaling, and others, affecting the level of glucose and lipid metabolism [30], which is of great significance for the study of metabolic diseases.

Through the analysis of miRNAs-mRNA network diagram, we found that miR-29 was significantly correlated with the regulation of JNK-1. miR-29 is important in a variety of biological processes, including cell proliferation, apoptosis, differentiation, and tumorigenesis [31]. The miR-29 family has been shown to regulate glycolipid metabolism, and its high expression is associated with elevated blood glucose and insulin resistance [32]. The mechanism of action of miR-29 includes inhibiting glucose transport and insulin receptor expression, promoting fatty acid synthesis, and inhibiting fatty acid β oxidation. Recent studies have verified a close relationship between miR-29 and AMPK signaling, and miR-29 can regulate retinopathy in diabetic mice by activating AMPK signaling [33]. AMPK is an important energy receptor that can be quickly activated by changes in intracellular energy states, such as decreased ATP level and increased AMP level; once activated, AMPK will regulate many downstream targets through phosphorylation [12]. Activation of AMPK signaling mainly promotes glycolipid catabolism and inhibits their synthesis. miR-29 can affect the activation and function of AMPK signaling in a few ways. First, miR-29 can directly regulate the expression of AMPK, studies have found that miR-29 can inhibit the translation and expression of AMPK mRNA by binding to the 3′-UTR region of AMPK mRNA; therefore, the increase of miR-29 will lead to the downregulation of AMPK, thus affecting the function of AMPK signaling. Second, miR-29 can also affect the function of AMPK signaling by regulating downstream molecules, these studies provide important clues of miR-29 in regulating energy metabolism. AMPK signaling is important in energy metabolism, glycolipid metabolism, protein synthesis and so on, and it is a key therapeutic target for many metabolic diseases [34]. Studies indicate that JNK-1 is activated in the AMPK signaling and involved in regulating AMPK. In addition, JNK-1 can also affect cell metabolism and growth via AMPK downstream signaling pathways, such as mTOR and p53. In addition, JNK-I signaling plays a pivotal role in metabolic regulation by interfering with insulin signaling, disrupting lipid metabolism, and impairing mitochondrial function. Activation of JNK-I is associated with insulin resistance, obesity, and metabolic disorders. AMPK, on the other hand, can directly phosphorylate JNK-1 and cause its activity to decrease. In summary, JNK-1 plays an important regulatory role in AMPK signaling, and this mutual relationship has an important biological significance in cell metabolism and disease.

Our study revealed that miRNA-29 affects the expression of key genes in glycolipid metabolism by regulating JNK-1; thus, it is important on the whole metabolic process, and this discovery provides a new idea for the research and treatment of metabolic diseases such as diabetes and NAFLD. Previous studies have shown that JNK-1 can inhibit AMPK signaling, thereby inhibiting fatty acid oxidation and glucose uptake. This study hypothesized that miRNA-29 targeted inhibition of JNK-1 may be through AMPK signaling, which not only revealed the important role of miR-29 in metabolism, but also provides new ideas for developing novel treatment strategies for obesity and metabolic diseases, and further research is needed to confirm that. We know that GLP-1RA plays an important role in both T2DM and NAFLD, so whether GLP-1RA is critical to interfere with miR-29 needs to be verified from further clinical and basic studies, which will provide new possibilities for the treatment of miR-29 in metabolic-related diseases such as diabetes, obesity, and cardiovascular diseases.
